# Case of Lithotripsy, Cured in Five Weeks from the First Sitting

**Published:** 1840-04-04

**Authors:** 

**Affiliations:** Attending Surgeon


### CLINICAL REPORTS.

### PENNSYLVANIA HOSPITAL.

*Case of Lithotripsy, cured in five weeks from the first sitting.* By Dr. RANDOLPH, Attending Surgeon.

[Reported by J. F. MEIGS, M. D. Resident Surgeon.]

J. E. aged 19, a native of the West Indies, was admitted into the Pennsylvania Hospital, under the care of Dr. Randolph, on the 30th January, 1840. He has been labouring for the last nine years under all the usual symptoms of calculus in the bladder. Upon landing in this country, about three months since, his sufferings were very much aggravated, and he was compelled to apply for medical aid. When first admitted into the hospital, the attacks of pain were very frequent and severe; he could not retain his urine longer than an hour at a time, and was, of course, obliged to rise often in the course of the night.

During the first week after his entrance, Dr. Randolph introduced frequently into his bladder, bougies, in order to accustom the urethra to their passage. He was directed to sit in warm water, when the attacks of pain were violent, and laudanum injections were given from time to time.

The following mixture was prescribed:—

℞.—Sodæ Bicarbonatis, ʒi.

Sacch. Albi.,

P. G. Acaciæ, āā ʒi.

Aquæ Fluv., ℥vi.

Ft. sol. S. c. m., d. in die.

*February 8th.*—At 10 A. M., twenty-five drops of laudanum were given, and the patient was directed to retain his urine, until half past eleven o'clock, when Dr. Randolph operated with Jacobson's instrument. The calculus, being of medium size, fell into the bas-fond of the bladder, and, in order to seize it, it was necessary to push it into the fundus, after which, there was not the slightest difficulty. The operation was attended with but little pain, the patient making no complaint whatever.

*Feb. 13th.*—The attacks of spasm have been less frequent and severe since the date of last note; has not, as yet, passed any fragments. Dr. Randolph again operated, and finding some difficulty with Jacobson's instrument, made use of Heurteloup's, with which he crushed it effectually, and with but little pain.

15*th.*—He passed out, yesterday, a considerable quantity of fine sand, but no large fragments. In the course of the day, a piece too large for expulsion, lodged in the membranous portion of the urethra, and was pushed back into the bladder, this morning, by means of a large sound. In the afternoon, a severe chill came on, followed by considerable fever. An anodyne enema was prescribed, with a tablespoonful of the following mixture to be taken every two hours.

℞.—Mist. Neutralis, ℥vi.
Ant. Tartaris, gr. i.
Ft. sol.

16*th.*—The fever has entirely disappeared; the skin is cool and soft.; pulse slow and regular. Mixture to be omitted.

23*d.*—Since the last note there have been no disagreeable symptoms. He has had no pain, excepting when the fragment of stone, before referred to, lodged in the urethra, and this was always readily removed by the introduction of a large sound. To-day the operation was repeated. The stone being rather small, lodges in the neck of the bladder, and, in consequence, the loop of Mr. Jacobson's instrument passes beyond it. Baron Heurteloup's was therefore preferred, and with this, the stone was seized four times successively, and crushed without the slightest difficulty to the operator, or pain to the patient.

*March* 1*st.*—Since the last note, considerable portions of stone have been voided, some of the size of peas. He has not suffered an attack of pain since the 25th. Instead of being obliged to rise frequently in the course of the night, he can now retain his urine for a space of six or eight hours. His general health is much improved.

Operation repeated to-day with the same instrument, and with like success.

Dr. Randolph operated also on the 7th and 10th of March. During this time, fragments of various sizes were constantly expelled, causing very little pain in their passage, though many of them were angular, with sharp edges.

On the morning of the 14th March, the patient declared himself cured, as he could feel no more stone in the bladder.

Having been sounded carefully by several physicians, it was declared by all that the calculus was entirely removed. He left the hospital in the course of a few days.

*Remarks.*—Dr. Randolph has usually preferred, in his operations for crushing calculi, the use of Mr. Jacobson's instrument. In cases, however, where the stone is small, as in the one above reported, and where it falls down into the bas-fond of the bladder, he has succeeded more readily and with less pain to the patient, by the use of Baron Heurteloup's. The difficulty which exists in such cases as the above, arises from the fact, that when Mr. J.'s instrument is introduced into the bladder and expanded, the loop passes over and beyond the stone, and it becomes necessary either to withdraw the instrument and push it into the superior fundus, or to turn the front edge of the loop downwards into the bas-fond to seize the calculus. This, when the instrument is of the size commonly used for adults, gives considerable pain, because, as the loop swells gradually from the shaft of the instrument it is necessary to withdraw it a little in order to dip it down behind the neck of the bladder, which causes considerable stretching of the prostatic portion of the urethra. In Heurteloup's, on the contrary, the jaws rise at nearly a right angle from the shaft, and may be turned downward without the same inconvenience as in Jacobson's.

